# Zinc glycine chelate ameliorates DSS-induced intestinal barrier dysfunction via attenuating TLR4/NF-κB pathway in meat ducks

**DOI:** 10.1186/s40104-023-00962-w

**Published:** 2024-01-19

**Authors:** Yaqi Chang, Ke Wang, Guangmang Liu, Hua Zhao, Xiaoling Chen, Jingyi Cai, Gang Jia

**Affiliations:** Institute of Animal Nutrition, Key Laboratory for Animal Disease-Resistance Nutrition of China, Ministry of Education, Sichuan Agricultural University, Chengdu, 611130 Sichuan China

**Keywords:** Cherry Valley ducks, Growth performance, Gut inflammation, Intestinal barrier, Zn-Gly

## Abstract

**Background:**

Zinc glycine chelate (Zn-Gly) has anti-inflammation and growth-promoting properties; however, the mechanism of Zn-Gly contribution to gut barrier function in Cherry Valley ducks during intestinal inflammation is unknown. Three-hundred 1-day-old ducks were divided into 5 groups (6 replicates and 10 ducks per replicate) in a completely randomized design: the control and dextran sulfate sodium (DSS) groups were fed a corn-soybean meal basal diet, and experimental groups received supplements of 70, 120 or 170 mg/kg Zn in form of Zn-Gly. The DSS and treatment groups were given 2 mL of 0.45 g/mL DSS daily during d 15–21, and the control group received normal saline. The experiment lasted 21 d.

**Results:**

Compared with DSS group, 70, 120 and 170 mg/kg Zn significantly increased body weight (BW), villus height and the ratio of villus to crypt, and significantly decreased the crypt depth of jejunum at 21 d. The number of goblet cells in jejunal villi in the Zn-Gly group was significantly increased by periodic acid-Schiff staining. Compared with control, the content of intestinal permeability marker D-lactic acid (D-LA) and fluxes of fluorescein isothiocyanate (FITC-D) in plasma of DSS group significantly increased, and 170 mg/kg Zn supplementation significantly decreased the D-LA content and FITC-D fluxes. Compared with control, contents of plasma, jejunum endotoxin and jejunum pro-inflammatory factors IL-1β, IL-6 and TNF-α were significantly increased in DSS group, and were significantly decreased by 170 mg/kg Zn supplementation. Dietary Zn significantly increased the contents of anti-inflammatory factors IL-10, IL-22 and sIgA and IgG in jejunum. Real-time PCR and Western blot results showed that 170 mg/kg Zn supplementation significantly increased mRNA expression levels of *CLDN-1* and expression of OCLN protein in jejunum, and decreased gene and protein expression of CLDN-2 compared with DSS group. The 120 mg/kg Zn significantly promoted the expressions of *IL-22* and *IgA*. Dietary Zn-Gly supplementation significantly decreased pro-inflammatory genes *IL-8* and *TNF-α* expression levels and TNF-α protein expression in jejunum. Additionally, Zn significantly reduced the gene and protein expression of TLR4, MYD88 and NF-κB p65.

**Conclusions:**

Zn-Gly improved duck BW and alleviated intestinal injury by regulating intestinal morphology, barrier function and gut inflammation-related signal pathways TLR4/MYD88/NF-κB p65.

**Supplementary Information:**

The online version contains supplementary material available at 10.1186/s40104-023-00962-w.

## Background

Ducks are reared worldwide due to their extensive adaptability and rapid growth [1]. However, in the context of antibiotic-free farming, a variety of environmental factors in commercial production can trigger intestinal inflammation, including high animal densities, intestinal pathogens (such as *Escherichia coli*), mycotoxins in feed and changes in feed formulation, leading to ducks suffering stress and bacterial endotoxin (ET) invasion, resulting in increased intestinal permeability and bacterial translocation [2, 3]. The resulting intestinal health problems lead to annual global economic losses of more than $6 billion [4]. Lipopolysaccharide (LPS) is one of the most common ETs in the gut, which can trigger the aggregation of macrophages by activating toll-like receptor 4 (TLR4) [5]. It also activates myeloid differentiation factor 88 (MYD88), which in turn activates nuclear factor κB (NF-κB) and tumor necrosis factor (TNF) receptor-related factor 6. These factors interact to stimulate the secretion of cytokines and chemokines, thereby accelerating occurrence of intestinal inflammation [6]. Therefore, there is an urgent need to find effective nutrients to promote intestinal barrier function and relieve intestinal inflammation of meat ducks.

The trace element zinc (Zn) plays an indispensable role in the maintenance of physiological functions such as metabolism, immune function, signal transduction and cell growth and differentiation [7]. As a structural factor, Zn regulates many protein functions, including transcription factors, enzymes, receptors and growth factors [8]. Organic and inorganic forms of Zn are used in the feed industry, and organic Zn is more easily absorbed than inorganic Zn. Studies showed that Zn glycine chelate (Zn-Gly) alleviated intestinal inflammation by modulating intestinal immunoglobulin gene expression [9]. In recent years, the view that Zn maintains the integrity of intestinal barrier function (physical, chemical, microbial and immune barriers) has been gradually confirmed. Sarkar et al. [10] found that Zn can regulate the phosphorylation of tight junction (TJ) proteins, and thus improve the intestinal barrier function. Shao et al. [11] showed that Zn enhanced the barrier function of intestinal epithelium by promoting the differentiation of Caco-2 cells and increasing expression of zonula occludens-1 (ZO-1). Levkut et al. [12] reported that addition of low-dose Zn significantly increased the expression of mucin 2 (MUC2) in the jejunum of broilers, and promoted the secretion of intestinal immunoglobulin A (IgA). He et al. [13] showed that Zn reduced the expression of interleukin (IL)-8 in intestinal cells of broilers suffering from necrotizing enteropathy, thus reducing the inflammatory response and improving intestinal barrier function. In addition, Zn is important for the maintenance and development of the innate and adaptive immune systems. Deficiency of Zn can lead to thymus atrophy, lymphocytopenia, impaired cellular and antibody-mediated immune responses, and even death in humans and animals [14, 15]. The Zn regulates the secretion of pro-inflammatory cytokines by activating or inhibiting NF-κB [16]. The underlying molecular pathways are based on TLR4/MYD88 signaling and are regulated by Zn signaling in monocytes [17]. Additionally, dextran sulfate sodium (DSS)-mediated, chemically-induced intestinal inflammation has been replicated in broiler chickens using oral gavage [18]. Increased intestinal permeability caused by DSS has been found in studies of broilers, as evidenced by leakage of fluorescein isothiocyanate dextran (FITC-D) into serum [18, 19]. In addition, elevated serum D-lactic acid (D-LA) levels were also observed in broilers fed DSS, suggesting damage to intestinal epithelial homeostasis caused by the toxic chemical DSS [20]. To the best of our knowledge, few studies have investigated the effects and mechanisms of Zn-Gly against intestinal inflammation of meat ducks.

Here, we selected DSS to establish the gut inflammation model of meat ducks in this study. Moreover, we studied the intestinal permeability and intestinal morphology in jejunum and measured inflammatory response and TJ protein related gene and protein levels following Zn-Gly supplementation and DSS challenge. Finally, we aim to investigate the beneficial effects and the underlying mechanisms of Zn-Gly action on the intestinal barrier function and inflammatory response when gut inflammation occurs in meat ducks.

## Materials and methods

### Ethics statement

Animals, diets and sampling procedures were approved by the Animal Welfare Committee of Sichuan Agricultural University (No. 20180718), Sichuan, China.

### Animal, diets and experimental design

Three-hundred 1-day-old male Cherry Valley ducks were randomly assigned to 5 treatments (each with 6 replicates and 10 ducks per replicate): non-challenged group (CON), DSS-challenged group (DSS) and DSS-challenged groups with 70, 120 or 170 mg/kg of Zn from Zn-Gly, respectively. The selected Zn doses were based on our previous results [21, 22]. The experiment lasted 21 d, and ducks were administered intragastrically with 2 mL of 0.45 g/mL DSS (molecular weight: 36,000–50,000, Cat: CD4421; Coolaber, Beijing, China) or the same amount of saline during d 15–21. The basal diet was formulated according to National Research Council guidelines [23], and the composition and nutrient levels are shown in Table 1. All birds were reared in cages (1 m × 0.75 m × 0.75 m) and allowed pellet feed and water ad libitum with adequate ventilation. In the first week, room temperature was maintained at 33 ± 1 °C, and then decreased by 3 °C every week. From the third weekend, the temperature was maintained at 24 ± 1 °C, relative humidity was 65%–75% and light was continuous. The Zn-Gly used in the experiment was provided by Chelota Group (Guanghan, China), with a Zn content of 21% and purity of 98.5%. The ducks for the experiment were purchased from Sichuan Mianying Breeding Duck Co., Ltd. (China).

**Table 1:** Ingredients and compositions of the basic diets, % (dry matter basis)

**Item**	**1–14 d**	**15–35 d**
Ingredients, %		
Corn	62.63	70
Soybean meal	28.3	23.12
Expanded soybean	5	3
Calcium carbonate	0.90	1
Dicalcium phosphate	1.9	1.86
NaCl	0.34	0.34
Choline chloride	0.15	0.15
Vitamin premix^a^	0.03	0.03
Mineral premix^b^	0.30	0.30
DL-Methionine	0.220	0.118
L-Lysine HCl	0.121	0.065
L-Threonine	0.072	0.017
L-Tryptophan	0.037	0
Total	100.00	100.00
Calculated nutrients, %		
Metabolizable energy, MJ/kg	12.32	12.35
Crude protein, %	20.06	17.52
Calcium, %	0.91	0.917
Non-phytate phosphorus, %	0.429	0.418
Digestible lysine, %	1.02	0.82
Digestible methionine, %	0.50	0.37
Zn, mg/kg	28.39	26.72

^a^Provided per kilogram of diet: vitamin A, 6,875 IU; vitamin D_3_, 1,640 IU; vitamin E, 30.01 mg; thiamine, 1 mg; riboflavin, 3.9 mg; pyridoxine, 3.375 mg; vitamin B_12_, 0.01 mg; calcium pantothenate, 8.85 mg; folate, 0.5 mg; biotin, 0.1 mg; niacin, 49.25 mg

^b^Provided per kilogram of diet: Cu (CuSO_4_∙5H_2_O), 8 mg; Fe (FeSO_4_∙7H_2_O), 80 mg; Mn (MnSO_4_∙H_2_O), 70 mg; Se (NaSeO_3_), 0.3 mg; I (KI), 0.4 mg

### Sample collection

At 21 days of the experiment, one duck of the average pen body weight (BW) was selected from each replicate and 30 ducks were anesthetized by intravenous injection with sodium pentobarbital (30 mg/kg BW) and slaughtered. The middle parts of duodenum and jejunum were collected 1–2 cm and placed in 4% paraformaldehyde solution for pathological section determination. In addition, jejunum tissues were collected in 2-mL cryo-storage tubes, immediately placed in liquid nitrogen and transferred to a −80 °C refrigerator for storage, for the detection of indicators related to intestinal inflammation and to intestinal barrier function.

At 21 days of the experiment, the ducks closest to the average BW in each treatment group were selected, and were administered as oral gavage with FITC-D (molecular weight: 3–5 kDa, Sigma-Aldrich, St. Louis, MO, USA) at 4.16 mg/kg BW. After 2 h, blood samples of ducks with FITC-D were collected.

### Measurements

#### Growth performance

On 1, 14 and 21 days of the experiment, fasting weight for each replicate was determined on the experimental ducks, and the feeding amount and residual amount were recorded. Then average daily gain (ADG), average daily feed intake (ADFI) and feed to gain ratio (F/G) were calculated according to the obtained data.

#### Morphological analysis

Fixed duodenum and jejunum tissues were dehydrated by ethanol gradient and paraffin-embedded. These sections were stained with hematoxylin and eosin (HE) according to standard protocol (Wuhan Servicebio Technology Co., Ltd., Wuhan, China). Intestinal sections stained with periodic acid-Schiff (PAS) were observed under an optical microscope (40× magnification). Goblet cells (purple) and neutral mucins were selected according to each visual field, and their numbers determined, and then organizational observations were made.

#### Plasma FITC-D and D-LA analysis

Plasma samples were centrifuged at 500 × *g* for 15 min, and then stored in a −20 °C refrigerator for later determination of intestinal permeability markers D-LA and FITC-D. Fluorescence levels of diluted plasma were analyzed as described by Vicuna et al. [24]. The detailed procedure for plasma FITC-D determination is shown in Fig. S1. The D-LA content was measured using an enzyme-linked immunosorbent assay (ELISA) (MM-91646O1; Jiangsu Meimian Industrial Co., Ltd., Jiangsu, China).

#### Measurements of inflammation factors and immunoglobulins in plasma and jejunum

Plasma samples were collected into sterile enzyme-free centrifuge tubes, and then the contents of ET (MM-1626O1), IL-1β (MM-925452O1), IL-6 (MM-91624O1), TNF-α (MM-32774O1) and D-LA (MM-91646O1) in plasma and jejunum and the concentrations of secretory immunoglobulin A (sIgA, MM-91217O1), immunoglobulin G (IgG, MM-91025O1), IL-10 (MM-33637O1), IL-22 (MM-925346O1) and MUC2 (MM-1790O1) in jejunum were measured by ELISA using a commercial kit (Jiangsu Meimian Industrial Co., Ltd.).

#### Quantitative real-time (qRT)-PCR analysis

The expression of TJ protein genes [claudin1 (*CLDN-1*), *CLDN-2*, *ZO-1*, *ZO-2* and occludin (*OCLN*)], genes associated with chemical barriers *MUC2*, *Notch1* and *Notch2* and immune-barrier-related genes *IgA* in the jejunum were analyzed using qRT-PCR according to Chang et al. [25]. Also, expression levels of inflammation-related genes were measured: *IL-8*, *TNF-α*, *IL-22*, *IgA*, *TLR4*, *MYD88*, *NF-κB* and *NLRP3*. Table 2 shows the sequence, product length and accession number of the primers used in this analysis. Briefly, RNA was extracted using RNA-easyTM Isolation Reagent (Vazyme, Nanjing, China) and reverse transcription, and qRT-PCR were conducted according to the protocol of commercial kits (Vazyme). The reference gene was *β-actin* and gene expression levels were calculated using the 2^–ΔΔCt^ method [26].

**Table 2:** Primer sequences for qRT-PCR

**Gene**	**Primer Sequence (5´→3´)**	**Size, bp**	**Accession No.**
*ZO-1*	Forward: ACGCTGGTGAAATCAAGGAAGAA Reverse: AGGGACATTCAACAGCGTGGC	255	XM_013093747.1
*ZO-2*	Forward: ACAGTGAAAGAAGCTGGCGTAG Reverse: GCTGTATTCCCTGCTACGGTC	131	XM_005019888.2
*OCLN*	Forward: CAGGATGTGGCAGAGGAATACAA Reverse: CCTTGTCGTAGTCGCTCACCAT	160	XM 013109403.1
*CLDN-1*	Forward: TCATGGTATGGCAACAGAGTGG Reverse: CGGGTGGGTGGATAGGAAGT	179	XM_013108556.1
*CLDN-2*	Forward: CTCCTCCTTGTTCACCCTCATC Reverse: GAACTCGCTCTTGGGTTTGTG	160	XM_005009661.2
*MUC2*	Forward: GGGCGCTCAATTCAACATAAGTA Reverse: TAAACTGATGGCTTCTTATGCGG	150	XM_005024513.2
*Notch1*	Forward: GTGAAATCGATGCGGACTGC Reverse: ATGAAGTCGGAGATGACGGC	148	XM_038164890.1
*Notch2*	Forward: CGCATCCGTGCTTGAACAAA Reverse: AGGGAGACCTGCTGCATAGA	123	XM_038182661.1
*IL-8*	Forward: GATTTCCGTGGCTCTGTCCC Reverse: CTCTGCGTCAGCTTCACATC	129	NM_001310420.1
*IL-22*	Forward: CTAAAATGGCCAGGGCCTCA Reverse: CGCCACCTCCTCAGTGTATG	179	XM_038167538.1
*TNF-α*	Forward: ACAGGACAGCCTATGCCAAC Reverse: ACAGGAAGGGCAACACATCT	165	XM_005019359.2
*IgA*	Forward: TCGCTCAAGGAACCCATCGT Reverse: GCGGGACCACGAGAACTTCA	174	U27222.1
*TLR4*	Forward: ACCCATTGTCACCAACATCATC Reverse: TGCCTCAGCAAGGTCTTATTCA	195	JN048668.1
*MYD88*	Forward: GGAGGATGGTGGTCGTCATT Reverse: CCGCAGGATACTTGGGAACT	158	NM_001310832.1
*NF-κB*	Forward: GCTGGCTAATTGGACCGACA Reverse: CAGGTCTGGCACGTATCTCG	122	XM_021271051.2
*β-actin*	Forward: AGAAATTGTGCGTGACATCAA Reverse: GGACTCCATACCCAAGAAAGAT	227	XM013108556.1

#### Immunofluorescence analysis

The expression of NF-κB p65 protein of jejunum was measured using immunofluorescence. After sectioning, tissue slides incubated with rabbit anti-NF-κB p65 (1:500, Cat: ET1603-12, HUABIO) antibody at 4 °C overnight. After washing three times for 5 min each with PBS, the slides were incubated with HRP goat anti-rabbit IgG (H + L) (1:300, ABclonal, AS014) secondary antibody for 60 min in darkness. Then, we use 4′-6-diamidino-2-phenylindole (Servicebio; G1012) for staining the nuclei.

#### Western blot (WB) analysis

Jejunal protein was extracted using radio immunoprecipitation assay lysis buffer (P0013B; Beyotime, Shanghai, China), and sample protein concentrations were determined by protein quantitative reagent kit-BCA method (23225; Thermo Fisher Scientific, Shanghai, China). Then the protein was isolated by 10% sodium dodecyl sulfate polyacrylamide gel electrophoresis and transferred onto 0.45-μm polyvinylidene fluoride (PVDF) membrane (Millipore, Eschborn, Germany) and blocked with Tris-buffered saline tween containing 5% nonfat milk at room temperature for 2 h. Then the PVDF membranes were incubated with primary antibodies overnight at 4 °C. The details for the primary antibodies are as follows: β-actin (1:10,000, Cat: AC026; ABclonal, Wuhan, China), anti-OCLN (1:500, Cat: 27260-1-AP; Proteintech Biotechnology, Chicago, IL, USA), TNF-α (1:500, Cat: 60291-1-Ig; Proteintech Biotechnology), anti-CLDN-1 (1:500, Cat: ER1906-37; HUABIO, Hangzhou, China), anti-CLDN-2 (1:500, Cat: 14085; ABclonal), IL-1β (1:1,000, Cat: 511369; ZenBio, Chengdu, China), TLR4 (1:1,000, Cat: 350146; ZenBio), MYD88 (1:500, Cat: 340629; ZenBio), NF-κB p65 (1:500, Cat: ET1603-12; HUABIO) and anti-phospho-NF-κB p65 (S529) (1:500, Cat: ET1604-27; HUABIO). Then, the PVDF membranes were incubated with secondary antibody HRP goat anti-rabbit IgG (H + L) (1:5,000; ABclonal; AS014) at room temperature for 2 h, and then BeyoECL Moon (P0018FS; Beyotime) reagent was used to observe the protein bands. Image Lab software (National Institutes of Health, DC, USA) was used to analyze the protein expression levels.

### Statistical analysis

The experimental data were preliminarily analyzed using Excel 2019, and the data were checked for normal distribution and equal variance using the Shapiro–Wilk and Levene’s tests before analysis, respectively. One-way analysis of variance was performed using SPSS 24.0 (SPSS Inc., Chicago, IL, USA) statistical software followed by Tukey’s post hoc test to determine statistical significance. Results were expressed as mean ± SEM, with *P* < 0.05 considered significant. GraphPad Prism 8.0 (GraphPad Inc., La Jolla, CA, USA) was used for data processing and graphics. In this study, indicators were measured by WB in 4 replicates, and other indicators were measured from 6 replicates.

## Results

### Effects of Zn-Gly on growth performance of ducks with DSS-induced intestinal inflammation

Table S1 shows the impact of Zn-Gly on growth performance of meat ducks at 14 d. There was no significant difference (*P* = 0.409) in the initial average BW of meat ducks among the five groups. Also, there were no significant differences in ADG and ADFI (*P* > 0.05), while the F/G of the 120 and 170 mg/kg Zn groups was significantly lower than the DSS group at 14 d (*P* < 0.05). Compared with the CON group, the BW and ADG of ducks in DSS group were significantly decreased (*P* < 0.05) at 21 d. Compared with the DSS group, dietary 70, 120 and 170 mg/kg Zn supplementation significantly increased BW at 21 d (*P* < 0.05), but there was no significant effect on ADFI during 15–21 d (*P* > 0.05). And compared with the CON group, DSS group significantly increased F/G of ducks. While the F/G of the 70, 120 and 170 mg/kg Zn group was significantly decreased during 15–21 d, compared with DSS group (Table 3).

**Table 3:** Effects of Zn-Gly on growth performance of meat ducks from 15 to 21 d

**Items**	**Dietary treatment**^**1**^					***P*-value**
	**CON**	**DSS**	**70 mg/kg Zn**	**120 mg/kg Zn**	**170 mg/kg Zn**	
BW (21 d), g	1,607.70 ± 12.61^a^	1,398.46 ± 25.49^c^	1,520.60 ± 31.94^b^	1,593.60 ± 18.00^ab^	1,623.46 ± 17.00^a^	< 0.01
ADG (15–21 d), g	103.54 ± 3.33^a^	79.72 ± 4.42^b^	95.83 ± 3.01^a^	101.11 ± 3.48^a^	103.44 ± 2.19^a^	0.007
ADFI (15–21 d), g	175.97 ± 2.85	166.39 ± 3.30	172.99 ± 5.27	184.86 ± 2.46	173.06 ± 7.71	0.171
F/G (15–21 d), g/g	1.70 ± 0.01^b^	2.09 ± 0.08^a^	1.80 ± 0.01^b^	1.83 ± 0.05^b^	1.67 ± 0.05^b^	0.001

Data represent mean values of six ducks per treatment

*BW* Body weight, *ADG* Average daily gain, *ADFI* Average daily feed intake, *F/G* Feed to gain ratio

^1^Dietary treatments were as follow: (1) control group (CON): basal diet; (2) DSS group (DSS): basal diet; (3) DSS + 70 mg Zn/kg from Zn-Gly; (4) DSS + 120 mg Zn/kg from Zn-Gly; and (5) DSS + 170 mg Zn/kg from Zn-Gly

^a–c^ The different lowercase letters indicate significant in variance analysis (*P* < 0.05)

### Effects of Zn-Gly on plasma ET and inflammatory cytokines in ducks with DSS-induced intestinal inflammation

**Fig. 1:**
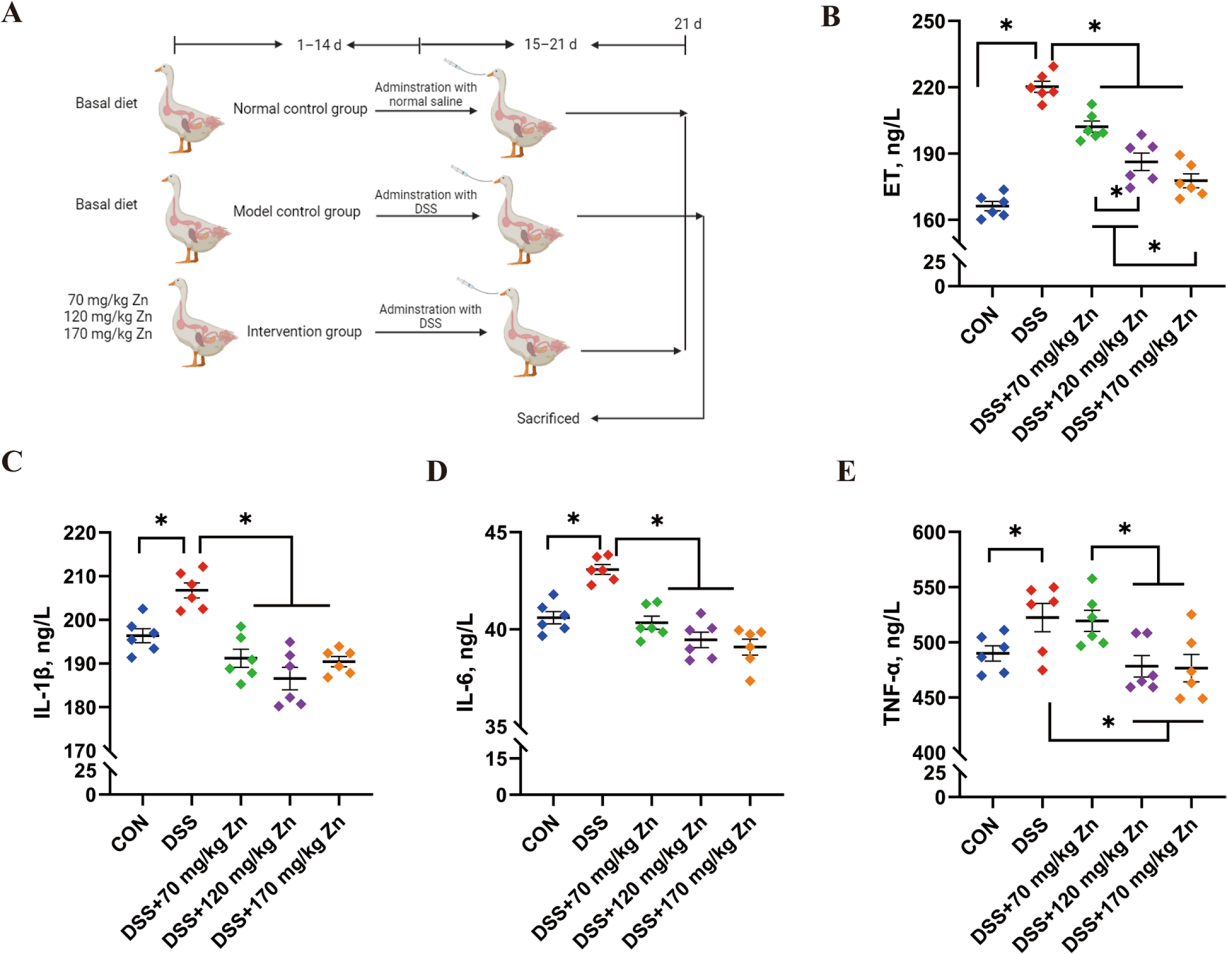
Establishment of intestinal inflammation model with DSS in meat ducks and effects of Zn-Gly on plasma ET and proinflammatory cytokines. **A** Establishment of DSS model of enteritis in duck. **B**–**E** The ET, IL-1β, IL-6 and TNF-α contents of plasma in ducks by the ELISA method. Mean ± SEM are shown (*n* = 6). ^*^P < 0.05

After DSS treatment, plasma levels of ET and pro-inflammatory cytokines IL-1β, IL-6 and TNF-α were significantly increased (*P* < 0.05, Fig. 1B–E). Different Zn-Gly concentrations were used to relieve the symptoms of enteritis in ducks, and levels of pro-inflammatory cytokines were significantly decreased by feeding Zn-Gly (*P* < 0.05).

### Effect of Zn-Gly on intestinal morphology of ducks with DSS-induced intestinal inflammation

**Fig. 2:**
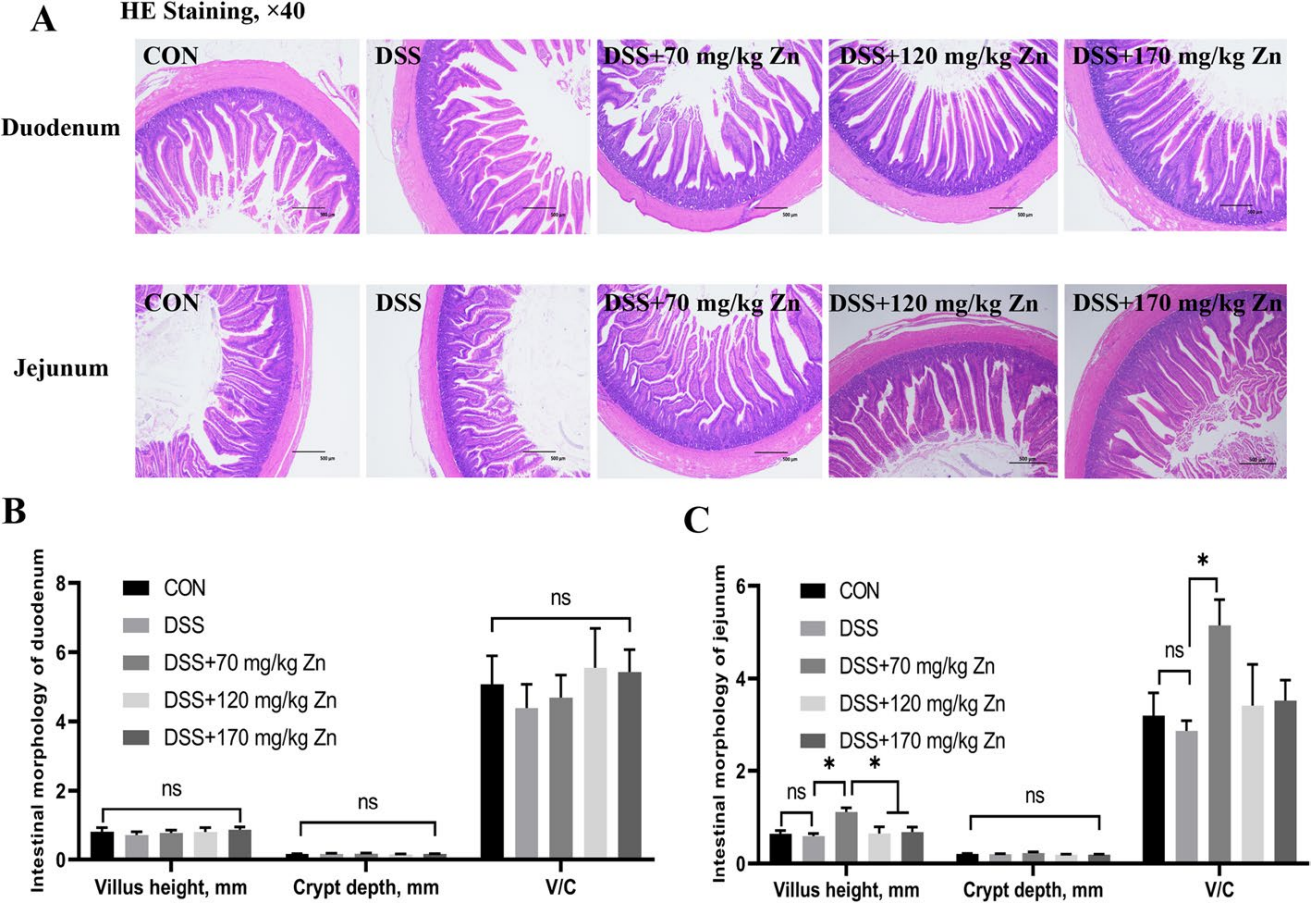
Effect of Zn-Gly on intestinal morphology of ducks with DSS-induced intestinal inflammation. **A** HE stained intestinal sections of duodenum and jejunum. **B** Villus height, crypt depth and V/C of duodenum. **C** Villus height, crypt depth and V/C of jejunum. Mean ± SEM are shown (*n* = 6). ^*^P < 0.05

Intestinal HE staining of duodenum and jejunum showed that DSS and Zn levels had no significant effect on duodenal villus height, crypt depth and villus height/crypt depth (V/C) (*P* > 0.05, Fig. 2). For the jejunum, the villus height, crypt depth and V/C in DSS group did not significantly differ from those in the CON group (*P* > 0.05), but villus height was significantly increased in the 70 mg/kg Zn group (*P* < 0.05).

### Effect of Zn-Gly on intestinal inflammatory cytokines in ducks with DSS-induced intestinal inflammation

**Fig. 3:**
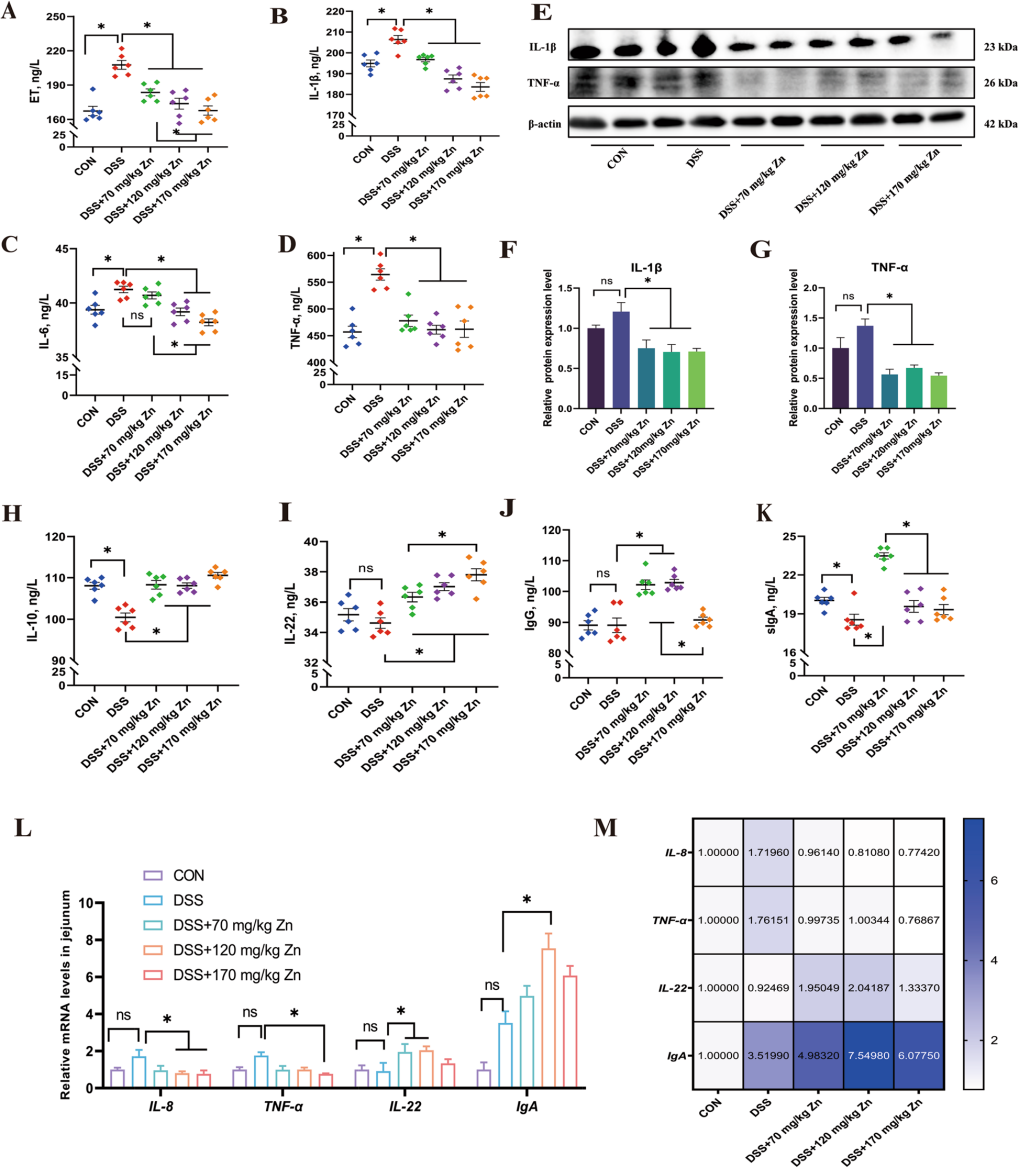
Effect of Zn-Gly on intestinal inflammatory cytokines in ducks with DSS-induced intestinal inflammation. **A–D** The ET, IL-1β, IL-6 and TNF-α contents of jejunum in ducks by the ELISA method (*n* = 6). **E**–**G** The relative protein expression of IL-1β and TNF-α in ducks (*n* = 4). **H**–**K** Contents of anti-inflammatory factors and immunoglobulins in jejunum of meat ducks (*n* = 6). **L** and **M** Relative expression of immune barrier related genes in jejunum of meat ducks (*n* = 6). Mean ± SEM are shown. ^*^P < 0.05

The DSS stimulation increased the levels of ET, IL-1β, IL-6 and TNF-α in jejunum tissue (*P* < 0.05), while Zn alleviated the increase of these pro-inflammatory cytokines (*P* < 0.05, Fig. 3A–E). Accordingly, results showed that protein expressions of IL-1β and TNF-α increased in jejunum of ducks treated with DSS, while Zn treatment significantly reverse their expression levels (*P* < 0.05, Fig. 3F and G). The levels of IL-10 and sIgA in the DSS group were significantly decreased (*P* < 0.05), while Zn supplementation reversed these indicators; notably, the sIgA level in the 70 mg/kg Zn group was significantly increased.

### Effects of Zn-Gly on intestinal barrier function in ducks with DSS-induced intestinal inflammation

#### Intestinal permeability

**Fig. 4:**
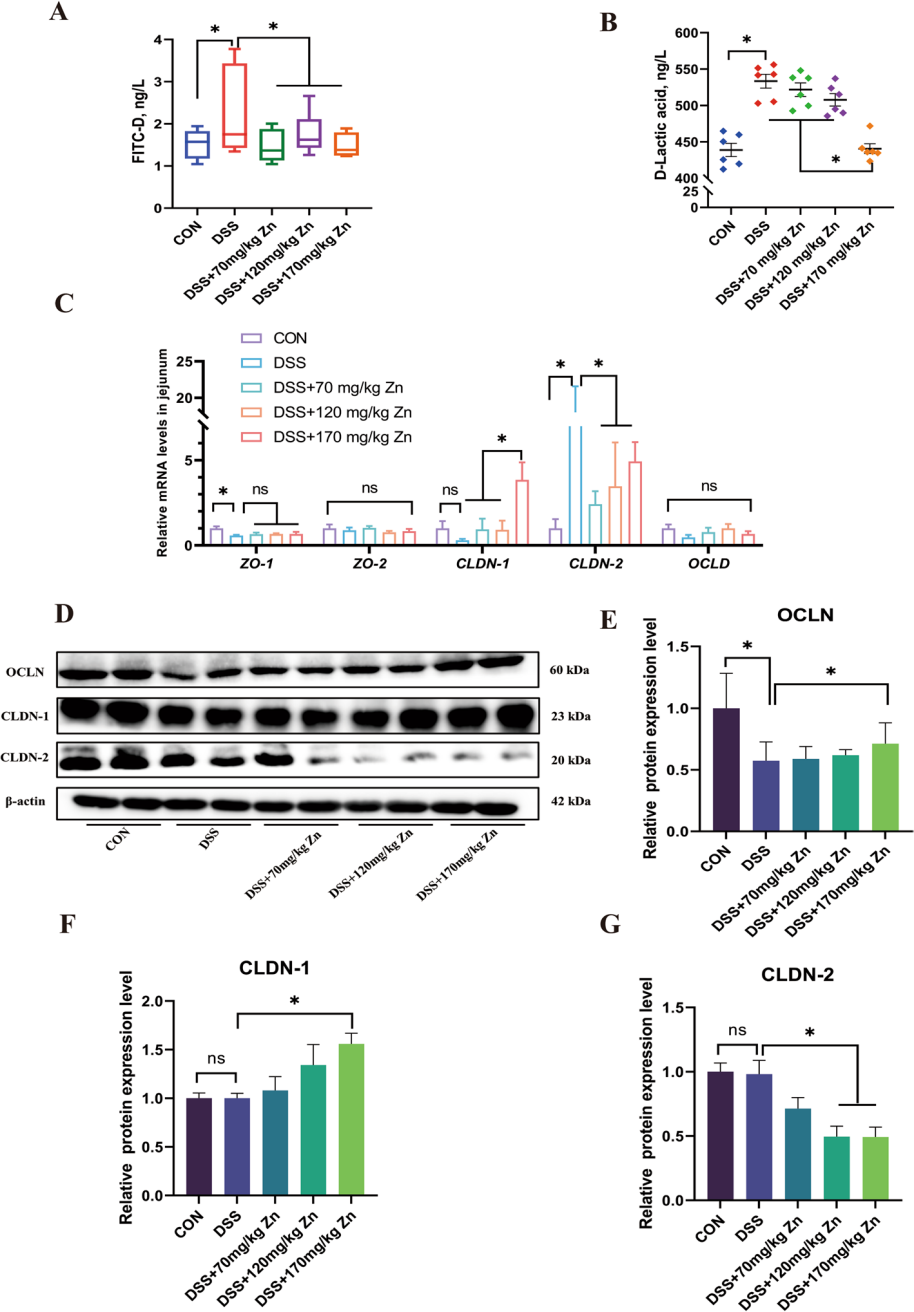
Effects of Zn-Gly on intestinal permeability and physical barrier function in ducks with DSS-induced intestinal inflammation. **A** and **B** The FITC-D and D-LA content in plasma of ducks (*n* = 6). **C** and **D** The relative expression of physical barrier-related genes (*n* = 6). **E**–**H** The relative protein expression of OCLN, CLDN-1 and CLDN-2 in ducks (*n* = 4). Mean ± SEM are shown. ^*^P < 0.05

The plasma FITC-D flux and D-LA content in the DSS group were significantly higher than those in the CON group (*P* < 0.05, Fig. 4A and B); however, both levels significantly decreased after Zn treatment (*P* < 0.05). Therefore, Zn may maintain the integrity of the intestinal barrier by regulating intestinal permeability.

#### Physical barrier

The TJ proteins play an important role in gut integrity, including ZO-1, OCLN, CLDN-1 and CLDN-2 [27]. Expression of genes *ZO-1* and *CLDN-2* were significantly affected in the DSS group (*P* < 0.05, Fig. 4C–G). The OCLN protein decreased significantly after DSS treatment, and expression levels of OCLN and CLDN-1 proteins were significantly higher after feeding Zn (*P* < 0.05). In addition, the mRNA levels of *CLDN-2* in jejunum tissues of DSS group were significantly increased, but significantly down-regulated gene and protein levels of CLDN-2 by Zn (*P* < 0.05).

#### Mucus barrier

**Fig. 5:**
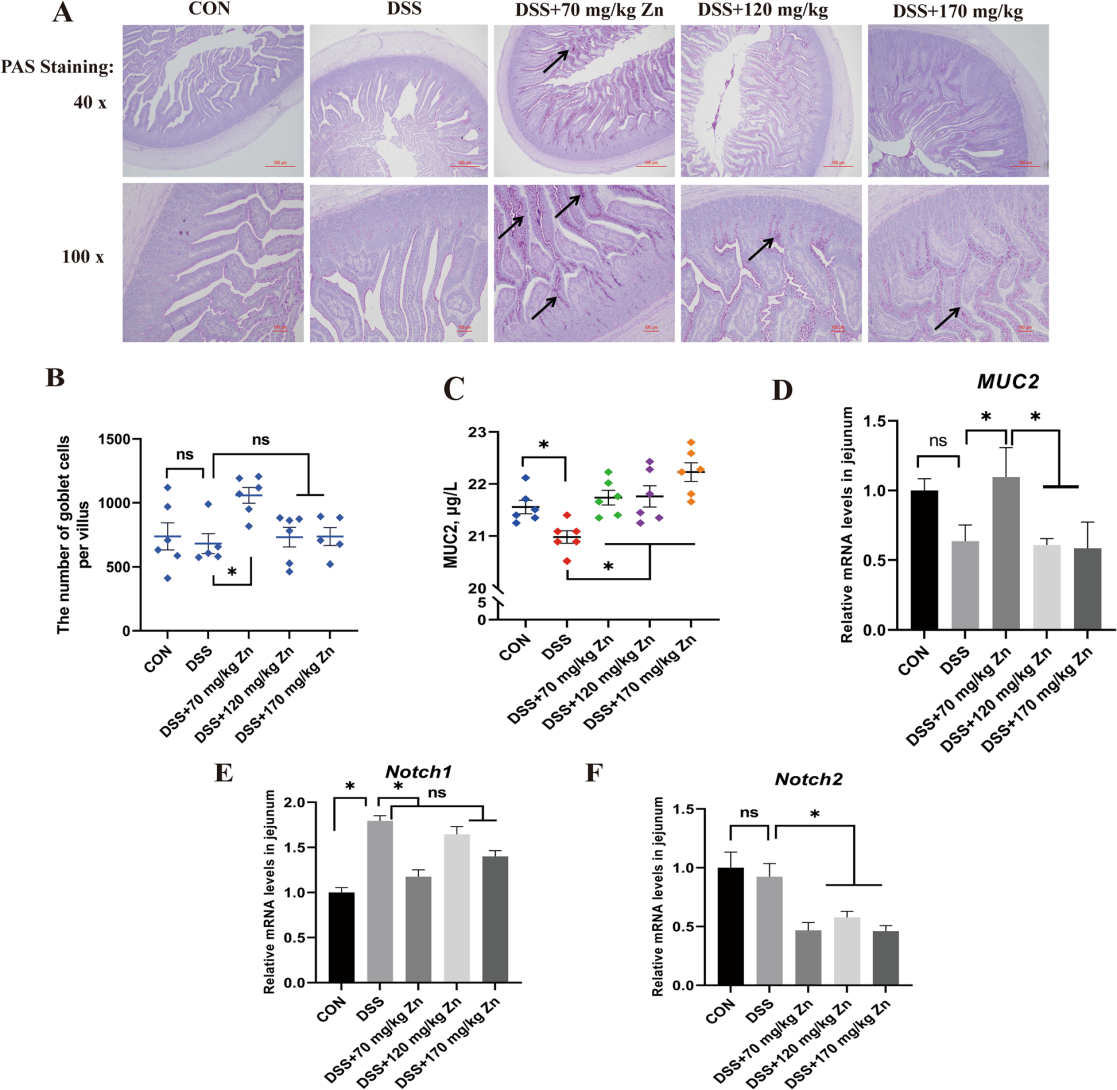
Effects of Zn-Gly on the number of goblet cells and chemical barrier function in ducks with DSS-induced intestinal inflammation. **A** PAS staining of jejunum. **B** The number of goblet cells per villus of jejunum in ducks. **C** Relative PAS-positive content of jejunum. **D** The MUC2 content of jejunum in ducks. **E**–**G** The relative gene expression of *MUC2*, *Notch1* and *Notch2* of jejunum in ducks. Mean ± SEM are shown (*n* = 6). ^*^P < 0.05

Generated by goblet cells, MUC2 is the major constituent of intestinal mucus layer [28]. Inhibition of the Notch pathway can result in a conversion of epithelial cells to goblet cells [29, 30]. Compared with CON, the number of goblet cells, and *MUC2* mRNA expression in jejunum of ducks with DSS-induced intestinal inflammation did not significantly differ (*P* > 0.05, Fig. 5A, B and D), but the concentrations of MUC2 was significantly decreased (*P* < 0.05, Fig. 5C). The above indicators were significantly improved after Zn-Gly treatment (*P* < 0.05). In addition, the expression level of *Notch1* mRNA associated with intestinal goblet cell differentiation was significantly increased after DSS treatment (Fig. 5E), while 70 mg/kg Zn significantly decreased the gene expression level (*P* < 0.05). The Zn-Gly treatment significantly decreased the gene expression level of *Notch2* in intestinal tissues (*P* < 0.05, Fig. 5F).

#### Immune barrier

**Fig. 6:**
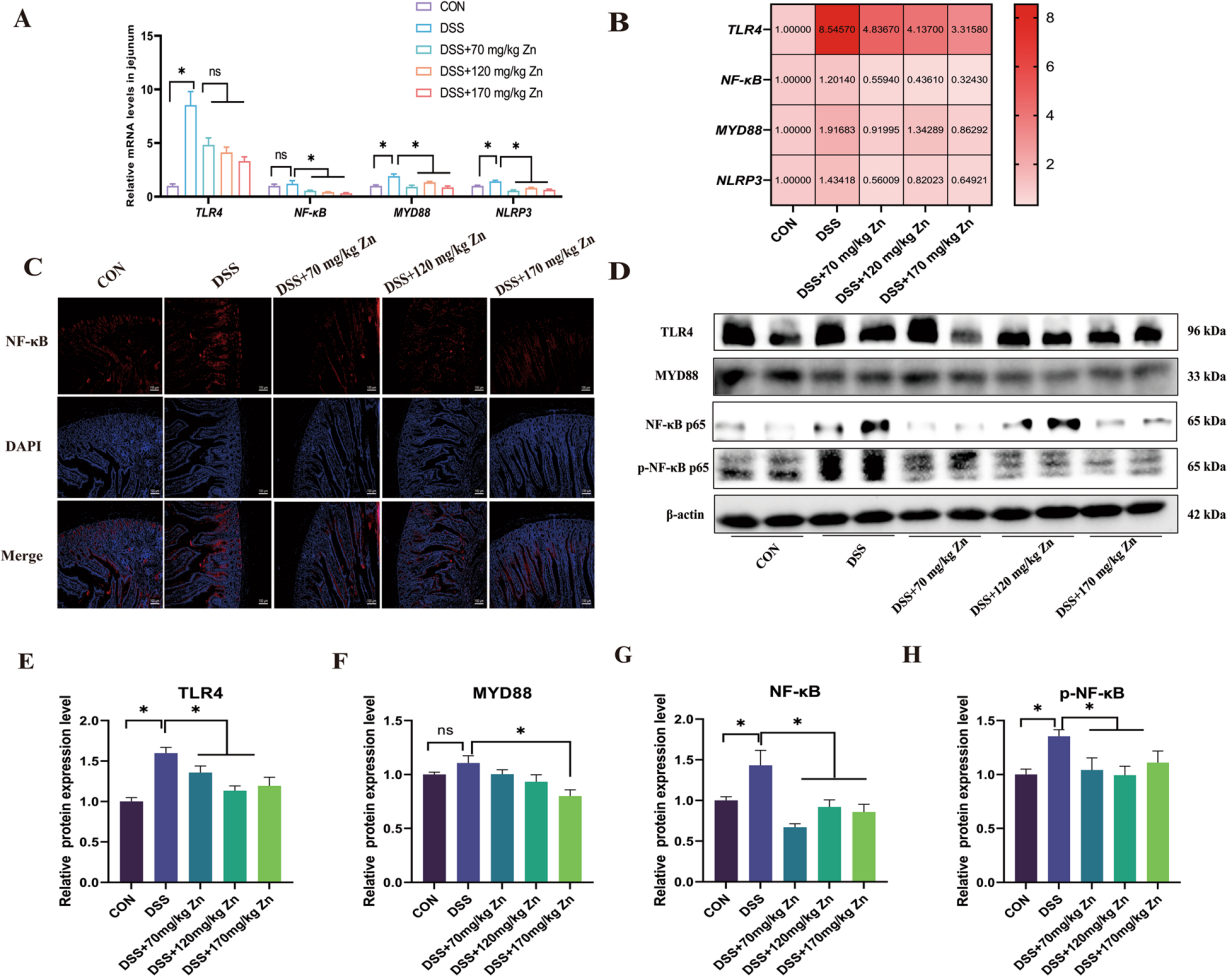
Effects of Zn-Gly on immune barrier function in ducks with DSS-induced intestinal inflammation. **A** and **B** The relative gene expression of *TLR4*, *NF-κB p65*, *MYD88*, *NLRP3* of jejunum in ducks (*n* = 6). **C** Immunofluorescence section of NF-κB p65 in jejunum. **D–H** The relative protein expression levels of TLR4, MYD88, p-NF-κB p65, NF-κB p65 of jejunum in ducks (*n* = 4). Mean ± SEM are shown. ^*^P < 0.05

Activation of TLR4/NF-κB signaling pathway is associated with the pathogenesis of inflammation [31]. Therefore, we further analyzed the impact of Zn-Gly on TLR4/NF-κB pathway (Fig. 6A–H). First, expression levels of genes *TLR4*, *MYD88* and *NLRP3* were significantly increased in the DSS group, while the mRNA levels of *NF-κB*, *MYD88* and *NLRP3* were significantly decreased by adding Zn-Gly (*P* < 0.05). Compared with CON, DSS treatment significantly increased TLR4, NF-κB p65 and phosphorylated NF-κB p65 (p-NF-κB p65) protein levels. The Zn significantly down-regulated the TLR4, MYD88, NF-κB p65 and p-NF-κB p65 proteins in jejunum treated with DSS (*P* < 0.05). These data suggested that Zn-Gly could improve intestinal barrier functions, possibly by inhibiting the TLR4/NF-κB signaling pathway.

## Discussion

In recent years, there has been considerable interest in exploring alternatives to antibiotics in animal production. There is ample evidence that trace element Zn can alleviate inflammatory response and promote intestinal barrier function [22, 32, 33]. The DSS impairs the intestinal epithelial barrier via stimulation of the secretion of pro-inflammatory cytokines that directly disrupt epithelial tight junctions. Firstly, clinical signs in murine models given oral DSS are a loss of BW along with intestinal histological lesions [34]. Vicuna et al. [24] also demonstrated that groups received DSS (0.75% in water for 5 d) had an increase in gut permeability associated with the serum levels of FITC-D. Furthermore, Zou et al. [20] explored the mechanism and revealed that increased inflammatory cytokine profiles measured in chickens with DSS could induce intestinal inflammation. These studies showed that the gut inflammation model measured by BW, intestinal morphology, FITC-D and inflammatory cytokine could be effective to study enteritis symptoms. In the current study, we measured the villus height, crypt depth and V/C of the jejunum, which in the DSS group did not significantly differ from those in the CON group; however, villus height and V/C had a decreasing trend, indicating that the DSS dose did not cause intestinal necrosis, but caused a significant decrease in BW and FITC-D and an increase in the content of pro-inflammatory cytokines, which can be used as a model of intestinal inflammation for further study. In this study, we analyzed the effect of organic Zn-Gly on a DSS-induced enteritis model of ducks and used the above indicators. These findings indicate that oral gavage with DSS caused non-necrotic enteritis, which could be developed as gut inflammation model in meat ducks.

In modern intensive feeding, ducks need a balanced nutrition strategy, especially the addition of trace elements. Although the amount of trace elements required by ducks is low, they play an important role in the enzyme system, physiology, metabolism, reproduction and growth of animals [35]. Among trace elements (copper, iron, manganese and Zn), Zn is important in stimulating piglet growth [36]. In poultry diets, Zn has long been used as a growth promoter and is used frequently due to its high bioavailability. In this study, there was significantly increased BW and ADG in meat ducks after Zn-Gly supplementation at 21 d, especially when 170 mg/kg Zn was added. Organic Zn may improve mineral absorption and reduce its binding to anti-nutritional factors such as phytic acid. However, we observed no effect of Zn-Gly on ADFI. In fact, in this study, DSS significantly reduced BW at 21 d and ADG during 15–21 d in meat ducks, and addition of Zn mitigated the loss of BW and ADG, with 170 mg/kg of Zn having the best effect. The ADFI of the DSS group was lower than that of other groups, but the difference was not significant. This showed that DSS treatment did not affect the feed intake of meat ducks. It is noteworthy that F/G of the DSS group was significantly higher than that of other treatment groups, demonstrating that the enteritis model was successfully constructed in the DSS group, and the feed conversion efficiency of meat ducks was affected. There was no significant difference in F/G of meat ducks between the control and the Zn supplementation groups, indicating that Zn may have a certain alleviating effect on enteritis of ducks. Therefore, Zn-Gly could reduce jejunum damage caused by DSS, restore intestinal barrier function and reduce gut permeability.

The effects of the organic Zn source Zn-Gly on intestinal health [37], tissue Zn deposition [38], antioxidant status [39], carcass traits [40] and body immune state [41] have been studied, suggesting its potential anti-inflammatory effect to promote intestinal health in animals. This study provided evidence of the protective effect of Zn-Gly on enteritis and explores the possible mechanism of its effect. A recent study demonstrated that Zn-Gly supplementation activated cellular and humoral immune responses in poultry, helping to maintain the balance between Th1 and Th2 responses and enhancing resistance to infection [41]. Studies have shown that IL-1β, IL-6 and TNF-α lead to intestinal barrier dysfunction and cell apoptosis [42]. In addition, Zn was reported to inhibit the production of IL-1β, IL-6 and TNF-α during LPS-induced damage to duck intestinal epithelial cells [43]. Therefore, we selected IL-1β, IL-6 and TNF-α as pro-inflammatory cytokines to further investigate the effect of DSS stimulation on inflammation. In the current study, Zn-Gly inhibited DSS-induced the production of pro-inflammatory IL-1β, IL-6 and TNF-α in plasma of meat ducks, suggesting that Zn-Gly alleviates intestinal inflammation via modulating inflammatory cytokine contents. In the meantime, contents of intestinal pro-inflammatory cytokines decreased following Zn-Gly supplementation, and the anti-inflammatory cytokines were increased. The results might indicate that the protective effects of Zn-Gly on intestinal inflammation were closely related to inhibiting production of pro-inflammatory cytokines and promoting production of anti-inflammatory cytokines.

Intact intestinal epithelial cells are the structural basis for maintaining normal barrier function. Overproduction of inflammatory cytokines erodes intestinal epithelial mucosa by destroying TJ proteins [44]. We found that Zn-Gly could reverse the decrease of *OCLN* and *CLDN-1* genes and protein expression induced by DSS, while the Zn-Gly group significantly decreased the *CLDN-2* gene and protein expression, suggesting that Zn-Gly directly protected the physical barrier of jejunum of ducks. Goblet cells protect against intestinal invasion by bacteria and pathogens by producing mucin [45]. Generated by goblet cells, MUC2 is the major macromolecular constituent of the intestinal mucus layer and is responsible for host defense [28]. An appropriate number of goblet cells are essential for MUC2 synthesis and maintaining the mucus layer. In contrast, dysfunctions in goblet cells promote inflammatory bowel disease [46, 47]. Studies have shown that *MUC2* deficiency exacerbates colon inflammatory responses and spontaneous colitis occurs in *MUC2* knockout mice [46]. In this study, PAS staining showed Zn-Gly restored goblet cell numbers and reversed DSS-induced downregulation of MUC2. Meanwhile, the Notch signaling pathway plays an important role in cell differentiation in the intestine. Inhibition of the Notch pathway in the intestinal epithelium results in a rapid and complete conversion of all epithelial cells to secretory cells such as goblet cells [29, 30]; consistent with this previous study, we found that 70 mg/kg Zn significantly inhibited *Notch1* and *Notch2* gene expression compared with the DSS group. In addition, enteritis caused by DSS also increased intestinal permeability, allowing inflammatory substances in the intestinal cavity to enter the circulation. Consistent with this, DSS ingestion resulted in significantly higher plasma ET, D-LA and FITC-D levels; and Zn-Gly reduced the D-LA and ET levels. In conclusion, Zn-Gly alleviated DSS-induced epithelial cell damage by enhancing the TJs, reducing intestinal permeability, decreasing intestinal inflammation and restoring intestinal barrier function.

Zhao et al. [48] showed that DSS-induced enteritis was associated with elevated IL-6, IL-1β and TNF-α levels as well as decreased levels of anti-inflammatory IL-10 in the gut. The Zn-Gly reduces intestinal inflammation response by regulating inflammatory cytokines. As previously noted, the DSS-induced increase in intestinal permeability allowed harmful substances to trigger an inflammatory response, activating the TLR4/NF-κB pathway [7]. In inflammatory responses, NF-κB is believed to be a key transcription factor that is activated and induces transcription of pro-inflammatory mediators. The cytokines trigger positive feedback regulation in inflammatory activation, ultimately damaging intestinal tissue [49]. In this study, Zn-Gly treatment reduced the protein expression of TLR4, MYD88, NF-κB p65 and p-NF-κB p65 in jejunum tissue, suggesting that Zn-Gly restore intestinal barrier function by reducing inflammation and inhibition the TLR4/ NF-κB p65 pathway.

## Conclusions

The Zn-Gly alleviated DSS-induced enteritis in ducks and restored intestinal barrier function by relieving inflammatory response and gut permeability. The underlying mechanism for the effect of Zn-Gly on the intestinal barrier protection may be regulated by repressing the TLR4/MYD88/NF-κB p65 signaling pathway. The present work provides a scientific foundation for Zn-Gly application in ducks.

### Supplementary Information

**Additional file 1:** **Fig. S1.** Procedure for intestinal permeability of FITC-D intragastric administration in meat ducks. **Table S1.** Effects of Zn-Gly on growth performance of meat ducks at 14 d.

## Data Availability

The original contributions presented in the study are included in the article, further inquiries can be directed to the corresponding author.

## Abbreviations

ADFI: Average daily feed intake
ADG: Average daily gain
BW: Body weight
CLDN1: Claudin1
CLDN2: Claudin2
D-LA: D-lactic acid
DSS: Dextran sulfate sodium
ET: Endotoxin
F/G: Feed to gain ratio
FITC-D: Fluorescein isothiocyanate
HE: Hematoxylin and eosin
IgA: Immunoglobulin A
IL-8: Interleukin 8
LPS: Lipopolysaccharide
MUC2: Mucin2
MYD88: Myeloid differentiation factor 88
NF-κB: Nuclear factor κB
OCLN: Occludin
PAS: Periodic acid-Schiff
p-NF-κB p65: Phosphorylated NF-κB p65
sIgA: Secretory immunoglobulin A
TJ: Tight junction
TLR4: Toll-like receptor
TNF-α: Tumor necrosis factor α
V/C: Villus height/crypt depth
Zn-Gly: Zinc glycine chelate
ZO-1: Zonula occludens-1
