# Supplementary material for: Zinc glycine chelate ameliorates DSS-induced intestinal barrier dysfunction via attenuating TLR4/NF-κB pathway in meat ducks
Source: J Anim Sci Biotechnol. 2024 Jan 19;15:5. doi: 10.1186/s40104-023-00962-w (PMC10797781; doi:10.1186/s40104-023-00962-w)
Supplement: Supplementary file 1 — Additional file 1: Fig. S1. Procedure for intestinal permeability of FITC-D intragastric administration in meat ducks. Table S1. Effects of Zn-Gly on growth performance of meat ducks at 14 d. [file 40104_2023_962_MOESM1_ESM.docx]

**Zinc glycine chelate ameliorates DSS-induced intestinal barrier dysfunction via attenuating TLR4/NF-κB pathway in meat ducks**


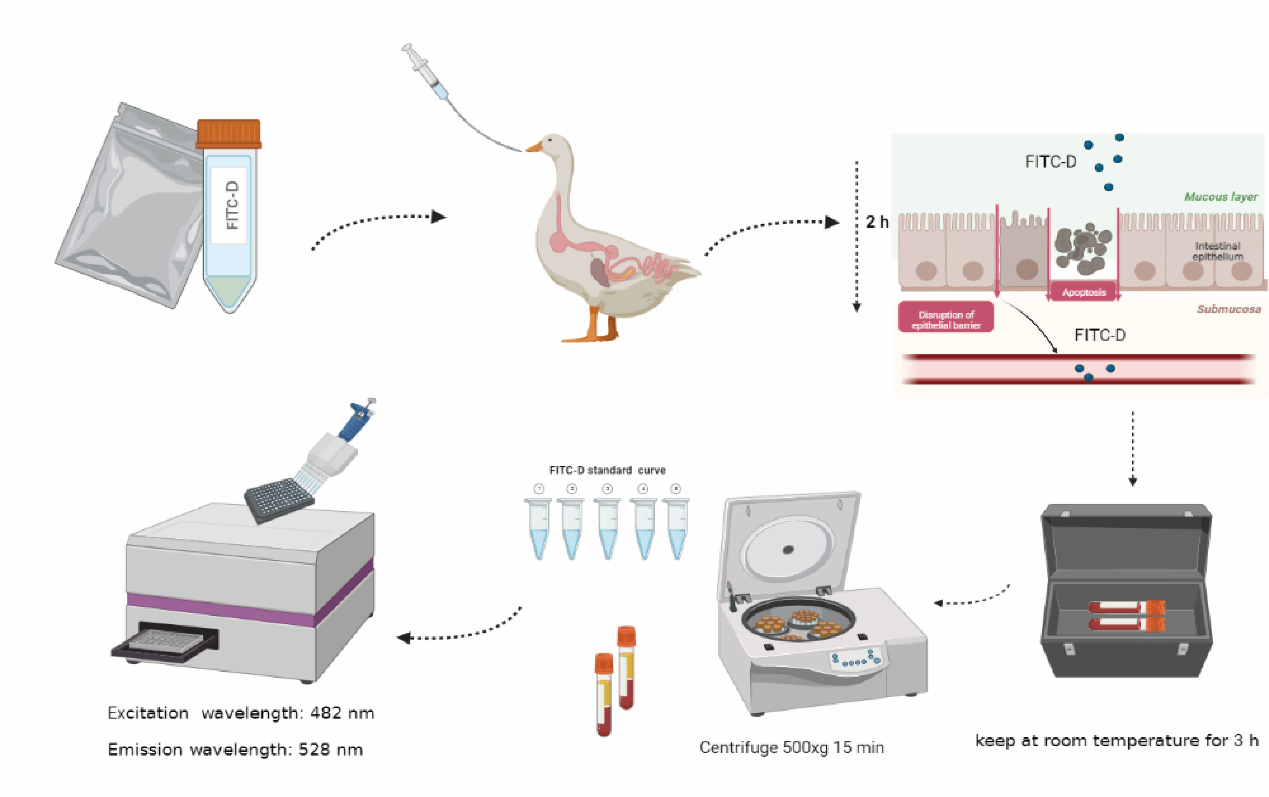


**Fig. S1** Procedure for intestinal permeability of FITC-D intragastric administration in meat ducks

**Table S1** Effects of Zn-Gly on growth performance of meat ducks at 14 d

| **Items** | **Dietary treatment^1^** | | | | | **ANOVA** |
| --- | --- | --- | --- | --- | --- | --- |
|  | **CON** | **DSS** | **70 mg/kg Zn** | **120 mg/kg Zn** | **170 mg/kg Zn** |  |
| BW, g |  |  |  |  |  |  |
| 1 d | 55.94 ± 0.20 | 56.27 ± 0.21 | 56.08 ± 0.14 | 56.23 ± 0.18 | 55.85 ± 0.15 | 0.409 |
| 14 d | 882.92 ± 12.61 | 840.42 ± 25.49 | 849.79 ± 31.94 | 885.83 ± 18.00 | 899.38 ± 17.00 | 0.295 |
| ADG, g |  |  |  |  |  |  |
| 1–14 d | 59.07 ± 0.90 | 56.01 ± 1.81 | 56.69 ± 2.29 | 59.26 ± 1.28 | 60.25 ± 1.21 | 0.289 |
| ADFI, g |  |  |  |  |  |  |
| 1–14 d | 87.94 ± 1.83 | 82.31 ± 3.13 | 81.22 ± 3.42 | 81.47 ± 1.61 | 81.76 ± 1.83 | 0.298 |
| F/G, g/g |  |  |  |  |  |  |
| 1–14 d | 1.49 ± 0.02^a^ | 1.47 ± 0.02^a^ | 1.43 ± 0.02^ab^ | 1.38 ± 0.02^b^ | 1.36 ± 0.02^b^ | < 0.0001 |

Data represent mean values of six ducks per treatment

*BW* Body weight, *ADG* Average daily gain, *ADFI* Average daily feed intake, *F/G* Feed to gain ratio

^1^Dietary treatments were as follow: (1) control group (CON): basal diet; (2) DSS group (DSS): basal diet; (3) DSS+70 mg Zn/kg from Zn-Gly; (4) DSS+120 mg Zn/kg from Zn-Gly; (5) DSS+170 mg Zn/kg from Zn-Gly

^a,b^The different lowercase letters indicate significant in variance analysis (*P* < 0.05)
